# Fatal metaldehyde poisoning in a dog confirmed by gas chromatography

**DOI:** 10.1186/s12917-020-02348-w

**Published:** 2020-05-15

**Authors:** Ana Flávia Machado Botelho, Amanda Milhomem Donato Machado, Rayanne Henrique Santana da Silva, Amanda Carvalho Faria, Lucas Santos Machado, Heloa Santos, Sandro de Melo Braga, Bruno Benetti Junta Torres, Marina Pacheco Miguel, Andrea Rodrigues Chaves, Marília Martins Melo

**Affiliations:** 1 Department of Veterinary Medicine, Universidade Federal de Goiás, Goiânia, Brazil; 2 Department of Analytical Chemistry, Goiânia, Goiás Brazil; 3 General Pathology Department, Instituto de Patologia Tropical e Saúde Pública, Goiânia, Goiás Brazil; 4 Department of Veterinary Clinic and Surgery, Universidade Federal de Minas Gerais, Belo Horizonte, Brazil; 5 Laboratório de Toxicologia Veterinária, Escola de Veterinária, Universidade Federal de Minas Gerais (UFMG), Avenida Antônio Carlos, 6627, CEP, Belo Horizonte, MG 31270-901 Brazil

**Keywords:** Intoxication, Snail bait, Toxicologic pathology, Forensic toxicology, Canine

## Abstract

**Background:**

Metaldehyde is a toxic pesticide used mainly as a molluscicide, responsible for intoxication and deaths in both humans and animals. Accidental exposure to metaldehyde in dogs is considered rare, but severe. Data concerning clinical and veterinary forensic toxicology are largely incomplete, especially regarding case reports in dogs. The present work reports a complete and detailed description of a case from the history, clinical evolution, pathological exams and toxicological diagnosis in an accidental case of metaldehyde poisoning in dog.

**Case presentation:**

An eleven-month-old, 3.0 kg, male German Spitz was presented for emergency care with acute vomiting and seizures 3 hours after suspected accidental ingestion of commercial molluscicide containing 3% metaldehyde (*Lesmax*®). The animal was in lateral recumbency and showed stuporous mentation, salivation, tonic-clonic status epilepticus, systemic tremors, bilateral miosis, absent palpebral, corneal, oculovestibular and gag reflexes, severely depressed spinal reflexes, dyspnea and tachycardia. Despite treatment, the patient progressed to comatose mentation and died. Necropsy examination revealed discrete lesions in the liver and central nervous system, while stomach examination revealed content of feed, activated charcoal and blue-green granules, compatible to the commercial formula of metaldehyde. Histology examination revealed extensive hemorrhage and severe centrolobular necrosis of the liver and tumefaction of Kupfer cells. Brain samples showed discrete hemorrhage and hyperemia. In order to confirm the diagnosis, samples from feces, stomach content, spleen, liver, heart, kidneys and brain were submitted gas chromatography analysis. Results confirmed the presence of metaldehyde in all samples. We describe clinicopathological abnormalities of a fatal case of metaldehyde poisoning in a dog, as well as postmortem diagnosis using gas chromatography.

**Conclusion:**

Metaldehyde poisoning is rarely reported, since the diagnosis is often difficult and the notifications scarce. To our knowledge, this is the first report describing clinical signs, pathological findings and chromatographic diagnosis. This report aims to contribute to the understanding of the pathogenesis of metaldehyde intoxication, to further explore veterinary forensic toxicology diagnosis.

## Background

Metaldehyde is a cyclic tetramer of acetaldehyde used for snail and slug baits, commonly found as blue-green granules or pellets, usually palatable o dogs due to the addition of bran or molasses. Concentration of such commercial products varies from 1.5 to 8% [1–3]. Metaldehyde supposedly kill slugs and snails through dehydration and paralysis [4], but the mechanism of metaldehyde toxicity in mammals is not clearly understood. Metaldehyde toxicosis has been reported in cats and dogs [5, 6]. Following oral exposure, metaldehyde is classified as category II acute toxicant (moderately toxic) in the United States [7]. This pesticide is cheap, extensively used worldwide and easily found in shops, which leads to accidental poisonings, especially in small children and pets [8].

Most previously reported studies regarding metaldehyde toxicity focused on incidence, epidemiology and clinical manifestations; however detailed clinical, pathological aspects and definitive diagnosis have not been reported. Intoxication reports indicate acute neurological signs and gastrointestinal abnormalities, including hyperesthesia, hyperthermia, severe muscle tremors, respiratory depression and seizures [5, 6, 9]. Diagnosis if often based on the history and clinical signs. However, definitive diagnosis is provided through chromatographic techniques such as High Performance Liquid Chromatography (HPLC) and Gas Chromatography (CG) [4, 10].

No antidotes are currently available, and treatment is mostly supportive, including decontamination procedures, anticolvulsive therapy and cardio-respiratory support [5, 10]. Here we describe detailed clinical evolution, post mortem, histopathological and toxicological findings of an accidental fatal case of metaldehyde poisoning in a dog.

## Case presentation

An eleven-month-old, 3.0 kg, male unsprayed German Spitz dog was presented for emergency care presenting acute vomiting and seizures 3 hours after suspected accidental ingestion of commercial molluscicide containing 3% metaldehyde (*Lesmax*®). Minutes after the alleged intoxication, the tutors administrated approximately 4 g of activated charcoal and a commercial “antitoxic” containing acetyl methionine, choline hydrochloride, cyanocobalamin, arginine hydrochloride, inositol, riboflavin, thiamine hydrochloride, pyroxene hydrochloride and nicotinamide.

The patient presented tachypnea, tachycardia (195 bpm), normal mucosae, hemoglobin saturation of 75% and rectal temperature of 40.5 °C. Nervous system alterations were observed: lateral recumbency with stuporous mentation, salivation, tonic-clonic status epilepticus, systemic tremors, bilateral miosis, oculovestibular and gag reflexes, severely depressed spinal reflexes.

Diazepam was administrated initially (1 mg/kg) intra rectal, followed by intravenous injection of 0.5 mg/kg with ringer lactate solution at 5 ml/kg/h rate. However, epileptic seizures continued and phenobarbitone was administrated (5 mg/kg). At the same time, oxygen was provided via intranasal catheter. Intravenous fluid therapy was maintained, followed by metamizole (25 mg/kg) and metroprolol (0.1 mg/kg) injections. Seizures were controlled, although tremors and miosis were still noticeable. Venous blood samples were collected for glycemia, hemogram and hemogasometry. Leucogram showed moderated leukopenia due to neutropenia and monocytopenia, and reactive lymphocytes, whilst other parameters remained normal. Hemogasometry showed a compensated metabolic acidosis, with bicarbonate reduction (6.8 mmol/L), base excess (− 15.5 mmol/L) associated with respiratory alkalosis, with carbon dioxide pressure at − 12.7 mmHg and discrete hyperkalemia (5.22mmmol/L). Glycemic levels were low and glucose was administrated at 0,5 g/kg.

During the epileptic seizures, the dog defecated and feces containing great amounts of blue grains compatible with metaldehyde were visualized. The patient progressed to comatose mentation and died approximately 4 hours after hospital admission.

**Fig. 1:**
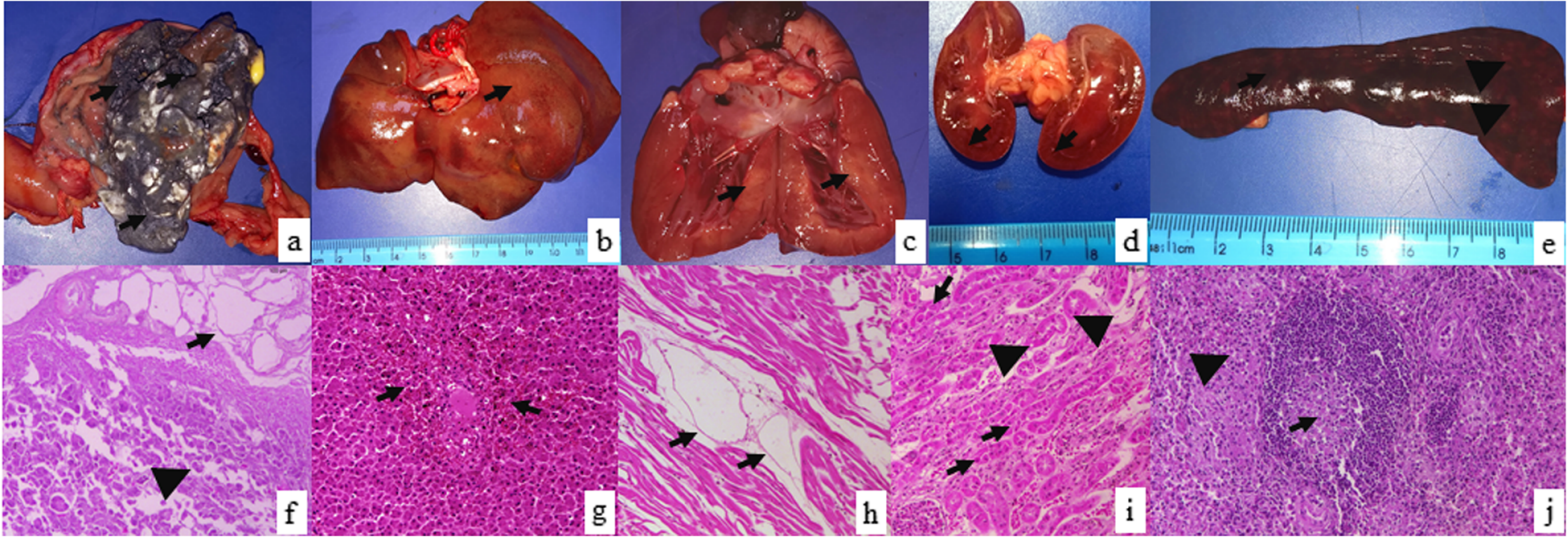
Gross lesions and histology findings of a dog accidently poisoned by metaldehyde. **a**. stomach lumen filled with black and dark blue granules(black arrow); **b**. liver showing multifocal white-yellowish areas (black arrow); **c**. multifocal pale areas on the myocardial tissue (black arrow); **d**. kidneys were diffusely brown red and had discrete white lines on the cortex (black arrow); **e**. spleen had multifocal white (black head arrow) and brown areas (blackarrow); **f**. pancreas with peripancreatic fat necrosis (black arrow) and multifocal necrosis of epithelial cells (black head arrow); **g**. liver showing centrolobular hepatic necrosis and hemorrhage (black arrow); **h**. heart showed dilation of lymphatic vessels (black arrow); **i**. kidney with multifocal vacuolization (black head arrow) and necrosis and desquamation of tubular cells (black arrow); **j**. spleen showed central white pulp necrosis (black arrow) and depletion of defense cells (black head arrow). (H&E stain, 400x)

Necropsy examination revealed moderate pale mucous, extensive organ congestion and tissue damage. Kidneys were diffusely brown red; liver had multifocal white-yellowish areas and moderate multifocal petechiae. The spleen was increased in size and the heart had multifocal pale areas on the myocardial tissue. Central nervous system was diffusely red while stomach examination revealed content of feed, activated charcoal and blue-green granules, compatible to the presented commercial formula of metaldehyde (Fig. 1). The remaining organs did not reveal macroscopic alterations.

Histology examination revealed extensive hemorrhage and severe centrolobular necrosis of the liver, tumefaction of Kupfer cells with multiple intracytoplasmic blue granules (Fig. 1). Brain samples showed multifocal discrete hemorrhage and hyperemia. Heart showed discrete hyperemia and extensive dilation of lymphatic vessels. Kidney samples showed multifocal epithelial cells degeneration, micro and macrovacuolization of tubular cells, necrosis and desquamation, intratubular eosinophilic material and severe diffuse hyperemia. Spleen analysis showed multifocal central white pulp necrosis and extensive hemorrhage and hyperemia. Stomach showed moderate and diffuse submucosa hyperemia, while pancreas revealed liquefactive multifocal necrosis and discrete necrosis of Langerhans cells with diffuse hyperemia.

At the necropsy, samples were collected from stomach, intestine, feces, spleen, kidneys, heart, liver and brain for toxicological analysis. The extraction process consisted in adding dichloromethane to each biological sample followed by vortex agitation for 1 min and ultrasound bath for 10 min. Supernatant was collected and prepared with nylon filters (0.22 μm) attached to syringes. Gas chromatography analysis were conducted using equipment Shimadzu GC-2010 Plus, column Restek Rtx-1 30 × 0.25 m (0.25 μm). The parameters used were: column equilibrium time 3 min, injection volume of 1 μL, detector temperature of 240 °C, sampler temperature of 100 °C, slit 1:50, gas from the mobile phase was helium, with 100 KPa of pressure, 15 min of total analysis and 5.2 min of retention time.

**Fig. 2:**
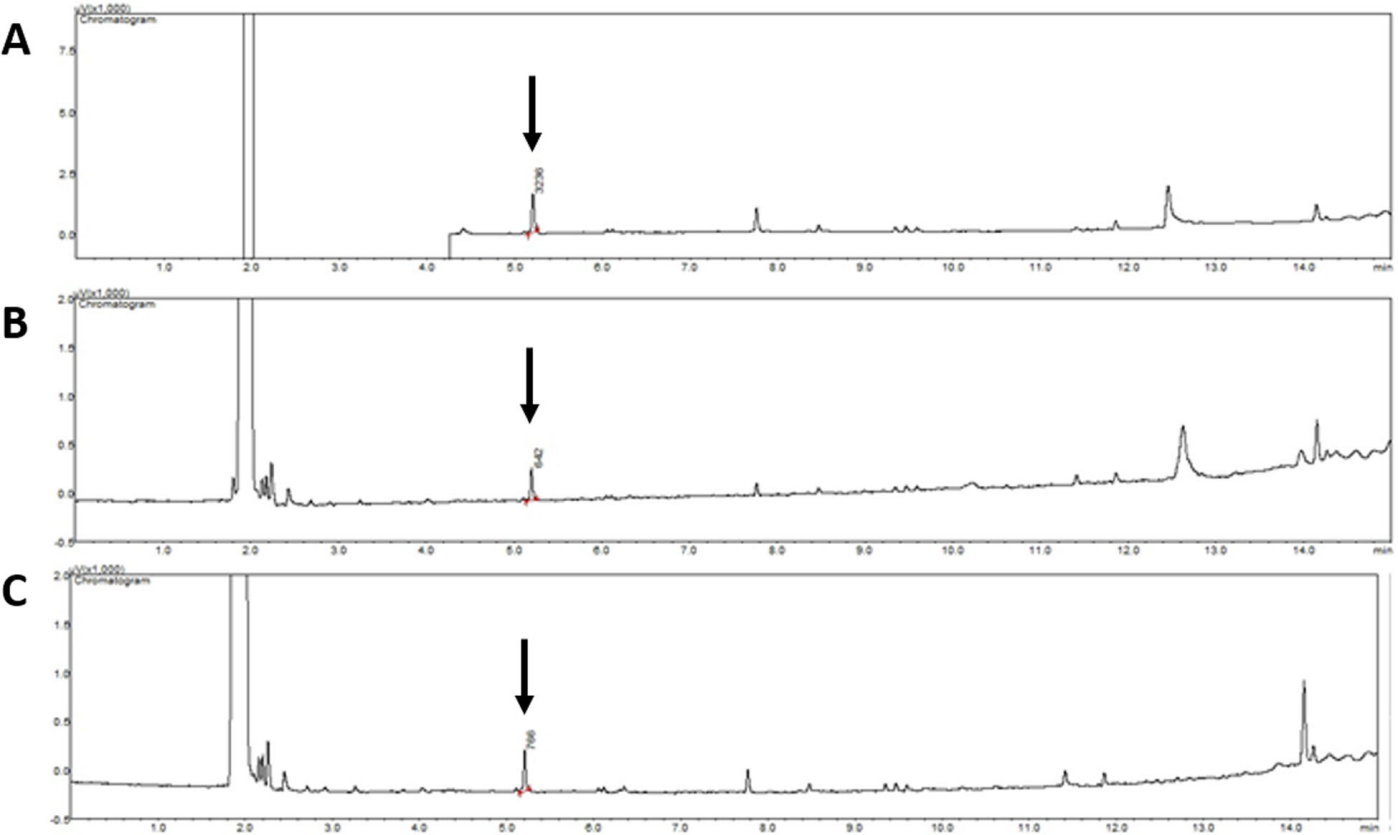
Chromatography analysis of a dog accidently poisoned by metaldehyde. Chromatograms of the stomach content (**a**), brain (**b**) and liver (**c**) revealing the presence of metaldehyde at 5.2 min retention time (red arrows)

For the calibration curve, commercial metaldehyde 3% (Lesmax, Insetimax®) was used with the same conditions as described above. Concentrations between 5 and 85 ppm were used to build the curve followed by the evaluation of the retention time and area under the curve. GC results confirmed the presence of metaldehyde in all samples: kidney, stomach content, feces, heart, spleen, liver, intestines, stomach and brain (Fig. 2).

## Discussion and conclusion

Metaldehyde is a readily available commercial pesticide, responsible for both intentional and accidental poisononing in animals. Data concerning clinical and veterinary forensic toxicology are largely incomplete, especially regarding case reports in dogs. The present work reports a complete and detailed description of a case from the history, clinical evolution, pathological exams and toxicological diagnosis in an accidental case of metaldehyde poisoning in dog.

Metaldehyde intoxication is considered a rare condition that causes severe clinical signs of neurotoxicity. Currently, the World Health Organization (WHO) classifies metaldehyde as a class II toxin and therefore it can be moderately hazardous with acute toxicity, although the mechanism involved in such toxicosis remains unknown [5]. It is estimated that once ingested metaldehyde is exposed to gastric secretions and partially converted to acetaldehyde and further oxidized into acetic acid, though the clinical relevance of this process is doubtful. Acetaldehyde may undergo enterohepatic circulation and both metaldehyde and acetaldehyde cross the blood brain barrier, resulting in severe neurotoxicity [11].

Patients with a history of ingestion usually present tremors, twitching, hyperpnoea, tachycardia, nystagmus, mydriasis, hypersalivation, ataxia, seizures, acidosis, hyperesthesia, diarrhea, dehydration, hyperthermia and death [2, 4, 5]. In this case, the most prominent presenting symptoms were tachypnea, stuporous mentation, systemic tremors and tonic-clonic status epilepticus. All these symptoms were consistent with previous reports of metaldehyde toxicity [2, 4, 5], although the mechanism of metaldehyde neurotoxicity is not fully understood, by it may result from both direct and indirect effects.

The present report showed that a few hours after the alleged ingestion, the patient quickly progressed to comatose state associated with metabolic acidosis. Metabolic acidosis with respiratory alkalosis has been previously reported, as metaldehyde may affect electrolyte and acid-base balances, which can cause acidosis that may also contribute to central nervous depression and hyperpnoea [2].

Blood samples may also reveal increase in creatine kinase (CK), aspartate aminotransferase (AST), lactate dehydrogenase (LDH), alanine aminotransferase (ALT), bilirubin, alkaline phosphatase (ALP) and hypoglycemia [5]. In the present report, blood samples were collected, but due to extensive hemolysis, blood count parameters were not evaluated.

There is no currently known antidote for metaldehyde toxicosis [12]. The main goals of the treatment include prevention of metaldehyde absorption, patient stabilization, management of the neurological signs and supportive care provision [3]. More recently, lipid emulsion therapy has shown potential in reverting severe clinical signs [13]. In the present case, conventional therapy was installed, but the gravity of the case and evolution of clinical signs impaired the success of the treatment. Gastrointestinal decontamination with activated charcoal is often recommend [5], but was not performed in this case due clinical instability and the prolonged time (3 h) before veterinary care.

Death is usually caused by central nervous system depression and respiratory failure. The possible cause of death in this case was the severe neurological depression, respiratory failure and shock, as previously reported [5] and confirmed by pathological findings.

Necropsy findings in patients with suspected metaldehyde poisoning are not pathognomonic and very few reports present histology findings. Hemorrhage and congestion found in the brain, heart and kidneys are compatible with acute hemorrhagic or neurologic chock. Centrolobular hepatic necrosis associated with hyperemia and hemorrhage are possibly related to the potential hepatotoxic effects of metaldehyde [5].

In order to confirm the intoxication, samples from feces, stomach content and different organs were submitted gas chromatography analysis. Results showed the presence of metaldehyde in all samples. Previous studies have detected intact metaldehyde in gastrointestinal tract, brain, blood and liver of orally dosed mice [2, 14], but to the authors knowledge this is the first report proving the presence of this pesticide in the feces, spleen, intestines, heart and kidney of an intoxicated dog.

In most cases of metaldehyde intoxication diagnosis is made through visual inspection, clinical signs and history [2, 4, 5]. However, this is not always possible, especially when reports are incomplete and when mastication or orally administered treatments, such as activated charcoal may distort the appearance of the granules. Gas chromatography analysis of necropsy tissues, including stomach content, feces and organs may be a useful tool in the diagnosis and should be considered as a laboratory tool in postmortem cases with suspected of metaldehyde intoxication.

Other causes of acute neurological signs and extensive liver damage that were not observed in the present report include lead, strychnine, ivermectin, carbamate, organophosphate, organochlorine intoxication, tremorgenic toxins and tricyclic antidepressant acute hepatitis [11]. These etiologies should also be considered as differential diagnoses for metaldehyde intoxication.

In conclusion, the present report describes a fatal case of metaldehyde intoxication in a dog. Clinical syndrome included tachycardia, tremors, seizures and metabolic acidosis. Pathological evaluation also revealed extensive liver damage and the presumed cause of death was neurological depression, respiratory failure and shock. To our knowledge, this is the first report proving the presence of this pesticide in feces, spleen, intestines, heart and kidney of an intoxicated dog. This report aims to contribute to the understanding of the pathogenesis of metaldehyde intoxication, to further explore veterinary forensic toxicology diagnosis.

## Data Availability

All relevant data are within this paper. The datasets generated during the current case study are available from the corresponding author on reasonable request.

## Abbreviations

HPLC: High Performance Liquid Chromatography
WHO: World Health Organization
CK: Creatine kinase (CK)
AST: Aspartate aminotransferase
LDH: Lactate dehydrogenase
ALT: Alanine aminotransferase
ALP: Alkaline phosphatase
GC: Gas Chromatography
LD50: Median lethal dose

## References

[1] Firth AM. Treatment of snail bait toxicity in dogs: retrospective study of 56 cases. Vet Emerg Critical Care. 1992. 10.1111/j.1476-4431.1992.tb00021.x.

[2] Puschner B. Metaldehyde. In: Peterson and Talcott, ed. Saunders company, Small Animal Toxicology. Philadelphia, 2001, p. 553–562.

[3] Richardson JA, Gwaltney-Brant SM, Huffman JD, Rosendale ME, Welch SL (2003). Metaldehyde toxicoses in dogs. Compend Cont Educ Pract.

[4] Booze TF, Oehme FW. An investigation of metaldehyde and acetaldehyde toxicities in dogs. Fundam Appl Toxicol. 1986. 10.1016/0272-0590(86)90217-4. 10.1016/0272-0590(86)90217-4 3084327

[5] Yas-Natan E, Segev G, Aroch I. Clinical, neurological and clinicopathological signs, treatment and outcome of metaldehyde intoxication in 18 dogs. J Small Anim Pract. 2007. 10.1111/j.1748-5827.2007.00360.x. 10.1111/j.1748-5827.2007.00360.x 17617165

[6] Studdert VP. Epidemiological features of snail and slug bait poisoning in dogs and cats. Aus Vet J. 1985. 10.1111/j.1751-0813.1985.tb14249.x. 10.1111/j.1751-0813.1985.tb14249.x 4062739

[7] World Health Organization (1996). Promotion of Chemical Safety Unit & Food and Agriculture Organization of the United Nations. WHO/FAO data sheet on pesticides. no.93.

[8] Bates NS, Sutton NM, Campbell A. Suspected metaldehyde slug bait poisoning in dogs: a retrospective analysis of cases reported to the veterinary poisons information service. Vet Rec. 2012. 10.1136/vr.100734. 10.1136/vr.100734 22859414

[9] Chiari M (2017). Pesticide incidence in poisoned baits: a 10-year report. Sci Total Environ.

[10] De Roma A, Miletti G, D’Alessio N, Rossini C, Vangone L, Galiero G, Esposito M. Metaldehyde poisoning of companion animals: a three-year retrospective study. J Vet Res. 2017. 10.1515/jvetres-2017-0041. 10.1515/jvetres-2017-0041 PMC5894430 29978088

[11] Khan SA. Differential diagnosis of common acute toxicologic versus nontoxicologic illness. Vet Clinics: Small Anim Prac. 2018. 10.1016/j.cvsm.2012.01.001. 10.1016/j.cvsm.2012.01.001 PMC7132484 22381187

[12] Beasley VR, Dorman DC. Management of toxicoses. Vet Clin North Am. 1990. 10.1016/s0195-5616(90)50027-3. 10.1016/s0195-5616(90)50027-3 2180180

[13] Lelescu CA, Muresan C, Muste A, Taulescu MA, Neagu AM, Nagy AL. Successful treatment of metaldehyde toxicosis with intravenous lipid emulsion in a dog. Acta Vet Brno. 2017. 10.2754/avb201786040379.

[14] Keller K, Shimizu G, Walter FG (1991). Olson KR Acetaldehyde analysis in severe metaldehyde poisoning. Vet Hum Toxicol.

